# Gender and Age Differences in Cardiac Size Parameters of Ghanaian Adults: Can One Parameter Fit All? Part Two

**DOI:** 10.4314/ejhs.v31i3.13

**Published:** 2021-05

**Authors:** Edmund Kwakye Brakohiapa, Benard Ohene Botwe, Benjamin Dabo Sarkodie

**Affiliations:** 1 Department of Radiology, University of Ghana school of Medicine and Dentistry; 2 Department of Radiography, University of Ghana School of Biomedical &Allied Health Sciences

**Keywords:** Cardiothoracic ratio, Transverse cardiac diameter, Chest radiograph

## Abstract

**Background:**

The cardiothoracic ratio (CTR) is a radiographic parameter commonly used in assessing the size of the heart. This study evaluated the gender and age-based differences in the average cardiothoracic ratios, and transverse cardiac diameters (TCD) of adults in Ghana.

**Method:**

Plain chest radiography reports of 2004 patients (without known chest related diseases) generated by two radiologists with at least 15 years' experience from July 2016 to June 2020 were retrospectively analyzed for this study. The CTR for each radiograph was calculated using the formula CTR=(TCD÷TTD)×100, where TCD and TTD represent transverse cardiac diameters and transverse thoracic diameters, respectively. Data were analyzed with the statistical package for social sciences version 23. The independent t-test and One-way Analysis of Variance tests were used in the analyses.

**Results:**

A total of 2004 patients' chest x-rays were used in the analyses. The ages of the patients ranged from 20–86 years old with a mean of 39.4±14.04 years. The mean CTR for males was 46.6 ± 3.7% while that of females was 47.7±3.7%. The difference in the overall CTR among the gender groupings was statistically significant (p = 0.001). There were statistically significant differences between the gender categories among patients in the following age groups: 30–39 (p=0.046), 40–49 (p=0.001), 50–59 (p=0.001) and 60–69 (p=0.001).

**Conclusion:**

The study reveals there are significant gender and age-related differences in cardiac size parameters obtained from routine, frontal chest radiographs. These differences, if considered, may result in early and appropriate treatment of cardiac pathology in some age groups.

## Introduction

The chest X-ray and electrocardiogram (ECG) are common, noninvasive methods of screening for heart disease. Due to its estimated sensitivity of 57% and negative predictive value of 83%, the cardiothoracic ratio (CTR) and chest radiograph are often not adequate for identifying the cause of cardiomegaly or microcardia (1). Studies have suggested that when used alone, the ECG had a lower predictive value, missing about 25% of patients with heart failure. The radiographic evaluation of the heart for disease therefore often requires complimentary investigation with an electrocardiogram or, especially with echocardiography in symptomatic patients (1). The chest X-ray is a quick, client-friendly, noninvasive screening method used in assessing the heart for disease, and the effect of management. The cardiothoracic ratio is a radiographic parameter commonly used in assessing the size of the heart (2). Heart disease may present with cardiomegaly (CTR > 50%), microcardia (CTR < 42%) or without an abnormality in shape or size (1,3). Others have suggested an upper limit of 55% for non-Caucasians, and 60% for the elderly (4). In resource poor environments such as Ghana, however, with staffing and logistics challenges, echocardiography services are not as readily available when compared to radiography services which had more than 300 radiography service providers distributed all over the country in 2014. Radiography in such a circumstance has been found to be highly comparable to echography, with a sensitivity of 85.7%, when the latter is used to measure the Left Ventricular Internal Dimension in diastole (LVIDd), and a sensitivity of 90.5% when compared to echocardiographic measurement of the Left Ventricular Internal Dimension in systole (5).

It is therefore important to obtain accurate radiographic information during screening or diagnostic examinations to complement ECG results, and inform the management path as minor differences in the CTR or transverse cardiac diameter may be pointers to ongoing heart disease (3). Early referral of diagnosed cardiac pathology can then be made to a cardiologist for early management, which is important in resource poor communities where people often present very late for medical treatment for various diseases. Clegg-Lamptey et al (6) researched to ascertain why some of their patients absconded during treatment in Ghana. Their study cited the high financial burden in medical care, and outmoded beliefs as reasons. High transportation cost for peri-urban and rural dwellers to capital cities where most specialist services are located, was also a reason why most of their patients absconded (6).

The CTR is obtained from the simple formula involving measurement of the transverse cardiac diameter (TCD) and transverse thoracic diameter (TTD) and relating them as follows: CTR=(TCD/TTD)X100. Though it is generally accepted that the normal upper limit of normal heart size is 50%, available literature states there are regional differences in CTR. Some authors have quoted 45% to 55% as mild cardiomegaly, greater than 55% as moderate to severe cardiomegaly and 44% or less as being normal (7). Studies in Ghana by Mensah et al showed that the average CTR for the Ghanaian population was 45.9 (8). Another Ghanaian study by Brakohiapa et al showed that the average CTR for adult Ghanaians was 46.6 for males and 47.8 for females (9). A study by Akosa and Armah showed that cardiomegaly contributed to 12.2% of an autopsy series conducted in Accra (the capital city of Ghana) over a 3-year period. The study also showed that hypertension made up 78.4% of the cause of cardiomegaly, with 47.8% of deaths from cardiomegaly occurring under the age of 50 years (10).

A pilot study conducted by our team between January 2012 and November 2013, with 1047 chest radiographs of asymptomatic individuals aged 20 – 80 years was presented at the 2^nd^ Annual General Scientific Meeting of the Ghana Association of Radiologists in 2014. The study showed significant differences in CTR between genders of the same age, and between age groups of the same gender (p-values = 0.001). We report the findings of our main study following the initial pilot stated above.

## Materials and Methods

**Study design and procedure**: Plain chest radiography reports of 2,004 asymptomatic patients generated by two radiologists with 15 years' experience from July 2016 to June 2020 were retrospectively analyzed for this study. Clinical information for each patient was extracted from their x-ray request forms presented at the x-ray department prior to having their x-rays taken. All reports of patients were generated from standard digital radiographs taken in posteroanterior (PA) position, at a film focus distance of 1.8m. Film exposures were made during an inspiratory breath-hold, with rib position on the 6^th^ rib anteriorly or the 10^th^ rib posteriorly. All images were interpreted from a view forum using Digital Imaging and Communications in Medicine (DICOM) software. The transverse cardiac diameters and transverse thoracic diameters were measured for all individuals using the system's electronic calipers. The cardiothoracic ratio for each radiograph was calculated using the formula: CTR=(TCD÷TTD)×100.

**Sample size determination**: The study sample size was determined using the Charan and Biswas' formula (11), where the proportion of such people in the populations was 87.8%, *d* is the absolute error of 5%, and a type error of 5% was considered. Accordingly, a sample size of 165 samples was estimated. However, to account for a better statistical outcome, a sample size of 2,004 was used.

**Inclusion and exclusion criteria**: The inclusion criteria for the study was all adults aged 20 to 89 years, who presented to the clinic for medical screening as a requirement for travel visa, employment, admission into a tertiary educational institution or for routine annual medical screening. The exclusion criteria included the presence of one or more of the following in a patient's clinical history: (i) cardiomegaly (defined in our study as a CTR>55%), (ii) upper lobe blood diversion (iii) pleural effusion, (iv) pulmonary artery enlargement, (v) lung consolidation, (vi) scoliosis/kyphosis, (vii) individuals younger than 20 years, (viii) individuals older than 89 years, (ix) a clinical history of hypertension, diabetes, cardiovascular disease and febrile illnesses. The equipment used was a GE Brivo XR385 digital radiography equipment (manufactured in March 2012, China).

**Data analysis**: Data handling was done by both descriptive and inferential analyses carried out using the statistical package for social sciences version 23. The independent t-test was used to identify whether there were significant differences between the mean CTD, CTR and TTD for both sexes. One-way Analysis of Variance (ANOVA) was carried out to identify whether there was a significant difference between the CTR, TTD and CTD among the various age groupings. Post-hoc tests (Bonferroni post-hoc tests) were used to demonstrate where the differences occur. Pearson's correlation test was used to determine association between age distribution and CTR, TTD and CTD among the various age groupings. A p-value of less than 0.05 was used to determine the significant level of the inferential analyses.

Ethical clearance was obtained from the University of Ghana College of Health Sciences Ethical and protocol Review Committee.

## Results

A total of 2004 patients' radiological images (PA chest x-rays) were used in the analyses. There were 1,053(53.0%) male and 951(47.0%) female data sets. The ages of the patients ranged from 20 – 86 years with a mean of 39.4 ± 14.04 years. The age distribution of the participants was as follows: 20–29 years (female = 301, 15%; male = 306, 15.1%), 30–39 years (female = 205, 10.2%; male = 288, 14.4%), 40–49 years (female = 167, 8.3%; male = 246, 12.3%), 50–59 years (female = 145, 7.2%; male = 144, 7.2%), 60–69 years (female = 98, 4.9%; male = 48, 2.4%), 70–79 years (female = 31, 1.6%; male = 18, 0.9%) and 80–89 years (female = 4, 0.2%; male = 3, 0.1%).

The CTR of all the participants ranged from 28.0% to 55.0%, with a mean of 47.1% ± 3.7. The mean CTR for males was 46.6% ± 3.7 while that of females was 47.7% ± 3.7. The difference in the overall CTR among the gender groupings was statistically significant (*p* = 0.001) (Table 1). There were statistically significant differences between the gender categories among patients in the following age groups: 30–39 (p=0.046), 40–49 (p=0.001), 50–59 (p=0.001) and 60–69 (p=0.001).

**Table 1:** CTR (cross-comparison) among gender distribution and age of patients

Age groups (years)	Gender	Mean CTR ±SD	*P*-value	Mean TCD ±SD	*P*-value	Mean TTD ±SD	*P*-value
20–29	Male	45.9 ± 3.5	0.839	13.4±1.2	0.001	29.1±1.7	0.001
	Female	45.9 ± 3.6		12.2±1.0		26.6±2.2	
30–39	Male	46.4±3.9	0.046	14.0±1.3	0.001	30.1±1.7	0.001
	Female	47.0 ± 3.6		12.9±1.4		27.3±2.1	
40–49	Male	47.1 ± 3.6	0.001	14.4±1.2	0.001	30.6±2.1	0.001
	Female	48.6 ± 3.2		13.4±1.0		27.5±1.5	
50–59	Male	47.4 ± 4.6	0.001	14.6±1.1	0.001	30.8±1.8	0.001
	Female	49.7 ± 3.9		13.6±0.9		27.5±1.6	
60–69	Male	47.0 ± 3.3	0.001	14.2±0.9	0.002	30.2±1.5	0.001
	Female	49.9. ± 2.9		13.6±1.0		27.4±1.5	
70–79	Male	47.4 ± 3.8	0.08	14.1±1.5	0.003	29.7±2.0	0.001
	Female	49.9 ± 3.0		13.0±0.9		26.4±1.4	
80–89	Male	47.6 ± 5.7	0.616	14.0±2.2	0.818	29.2±1.9	0.498
	Female	49.6 ± 4.8		13.7±1.2		28.7±3.2	
All groups	Male	46.6 ± 3.7	0.001	14.0±1.3	0.001	30.0±1.9	0.001
(20–89)	Female	47.7 ± 3.7		12.9±1.2		27.1± 2.0	

SD = Standard deviation; TCD =transverse cardiac diameter; TTC = transthoracic diameter

**Figure 1:**
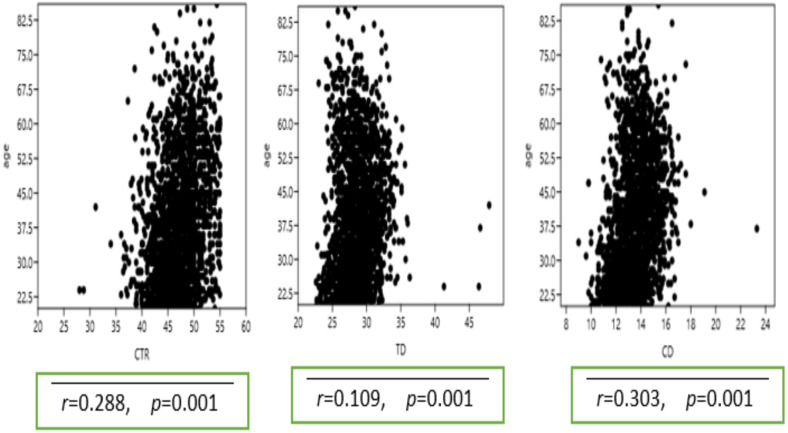
Associations between age distribution and CTR, TCD and TTD (in order from left to right) for the entire patients

The mean TCD and TTD measurement for all the patients were 13.5cm±1.4 (range 9.0–23.3) and 28.7cm ± 2.4 (range 22.6–47.9), respectively. A mean TCD of 14.0±1.3 was observed in the male sample, while the female sample had a mean TCD of 12.9±1.2. The male and female participants had a mean TTD of 29.1cm ± 1.7 and 26.6cm ± 2.2, respectively. Apart from the 80–89-year group, all the other age groupings demonstrated statistically significant differences among the gender groups in terms of TCD and TTD. Details of these findings are presented in Table 1. A statistically significant association between the participants' age and CTR, TCD and TTD was also observed (Figure 1).

Tables 2–5 further present results of cross comparison of CTR and TCD among age and gender groups.

**Table 2:** Cross comparison of CTR among age groups of males

(I) Age (yrs)	(J) Age (yrs)	Mean Difference (I-J)	Std. Error	*p*-value	95% Confidence Interval for Difference	

					Lower Bound	Upper Bound
20s	30s	-.441	.297	1.000	-1.347	.465
	40s	-1.215*	.310	.002	-2.159	-.270
	50s	-1.503*	.366	.001	-2.618	-.388
	60s	-1.090	.562	1.000	-2.803	.622
	70s	-1.510	.878	1.000	-4.186	1.165
	80s	-1.655	2.101	1.000	-8.055	4.745
30s	20s	.441	.297	1.000	-.465	1.347
	40s	-.774	.314	.295	-1.731	.184
	50s	-1.062	.370	.087	-2.188	.064
	60s	-.649	.565	1.000	-2.369	1.070
	70s	-1.069	.880	1.000	-3.750	1.611
	80s	-1.214	2.102	1.000	-7.616	5.188
40s	20s	1.215*	.310	.002	.270	2.159
	30s	.774	.314	.295	-.184	1.731
	50s	-.288	.380	1.000	-1.446	.869
	60s	.124	.572	1.000	-1.616	1.865
	70s	-.296	.884	1.000	-2.989	2.398
	80s	-.440	2.104	1.000	-6.848	5.967
50s	20s	1.503*	.366	.001	.388	2.618
	30s	1.062	.370	.087	-.064	2.188
	40s	.288	.380	1.000	-.869	1.446
	60s	.413	.604	1.000	-1.426	2.251
	70s	-.008	.906	1.000	-2.765	2.750
	80s	-.152	2.113	1.000	-6.587	6.283
60s	20s	1.090	.562	1.000	-.622	2.803
	30s	.649	.565	1.000	-1.070	2.369
	40s	-.124	.572	1.000	-1.865	1.616
	50s	-.413	.604	1.000	-2.251	1.426
	70s	-.420	1.001	1.000	-3.469	2.629
	80s	-.565	2.156	1.000	-7.129	6.000
70s	20s	1.510	.878	1.000	-1.165	4.186
	30s	1.069	.880	1.000	-1.611	3.750
	40s	.296	.884	1.000	-2.398	2.989
	50s	.008	.906	1.000	-2.750	2.765
	60s	.420	1.001	1.000	-2.629	3.469
	80s	-.144	2.259	1.000	-7.024	6.735
80s	20s	1.655	2.101	1.000	-4.745	8.055
	30s	1.214	2.102	1.000	-5.188	7.616
	40s	.440	2.104	1.000	-5.967	6.848
	50s	.152	2.113	1.000	-6.283	6.587
	60s	.565	2.156	1.000	-6.000	7.129
	70s	.144	2.259	1.000	-6.735	7.024

* The mean difference is significant at the .05 level.

Dependent Variable is CTR, yrs = years

**Table 3:** Cross comparison of CTR among age groups of females

(I) Age (yrs)	(J) Age (yrs)	Mean Difference (I-J)	Std. Error	*p*-value	95% Confidence Interval for Difference	

					Lower Bound	Upper Bound
20s	30s	-1.183*	.303	.002	-2.104	-.261
	40s	-2.766*	.322	.000	-3.748	-1.784
	50s	-3.860*	.338	.000	-4.889	-2.832
	60s	-4.035*	.389	.000	-5.219	-2.852
	70s	-3.337*	.630	.000	-5.257	-1.417
	80s	-3.772	1.681	.528	-8.894	1.351
30s	20s	1.183*	.303	.002	.261	2.104
	40s	-1.583*	.348	.000	-2.644	-.522
	50s	-2.678*	.363	.000	-3.782	-1.573
	60s	-2.853*	.410	.000	-4.103	-1.603
	70s	-2.154*	.644	.018	-4.115	-.193
	80s	-2.589	1.687	1.000	-7.727	2.549
40s	20s	2.766*	.322	.000	1.784	3.748
	30s	1.583*	.348	.000	.522	2.644
	50s	-1.095	.379	.084	-2.250	.061
	60s	-1.270	.425	.061	-2.565	.025
	70s	-.571	.653	1.000	-2.561	1.419
	80s	-1.006	1.690	1.000	-6.155	4.143
50s	20s	3.860*	.338	.000	2.832	4.889
	30s	2.678*	.363	.000	1.573	3.782
	40s	1.095	.379	.084	-.061	2.250
	60s	-.175	.437	1.000	-1.506	1.156
	70s	.523	.661	1.000	-1.490	2.537
	80s	.089	1.693	1.000	-5.069	5.247
60s	20s	4.035*	.389	.000	2.852	5.219
	30s	2.853*	.410	.000	1.603	4.103
	40s	1.270	.425	.061	-.025	2.565
	50s	.175	.437	1.000	-1.156	1.506
	70s	.698	.688	1.000	-1.399	2.796
	80s	.264	1.704	1.000	-4.928	5.455
70s	20s	3.337*	.630	.000	1.417	5.257
	30s	2.154*	.644	.018	.193	4.115
	40s	.571	.653	1.000	-1.419	2.561
	50s	-.523	.661	1.000	-2.537	1.490
	60s	-.698	.688	1.000	-2.796	1.399
	80s	-.435	1.775	1.000	-5.842	4.972
80s	20s	3.772	1.681	.528	-1.351	8.894
	30s	2.589	1.687	1.000	-2.549	7.727
	40s	1.006	1.690	1.000	-4.143	6.155
	50s	-.089	1.693	1.000	-5.247	5.069
	60s	-.264	1.704	1.000	-5.455	4.928
	70s	.435	1.775	1.000	-4.972	5.842

* The mean difference is significant at the .05 level.

Dependent Variable is CTR, yrs = years

**Table 4:** Cross comparison of TCD among age groups of males.

(I) Age (yrs)	(J) Age (yrs)	Mean Difference (I-J)	Std. Error	*p*-value	95% Confidence Interval for Difference	

					Lower Bound	Upper Bound
20s	30s	-.599*	.102	.000	-.910	-.288
	40s	-1.035*	.107	.000	-1.360	-.711
	50s	-1.227*	.126	.000	-1.610	-.844
	60s	-.833*	.193	.000	-1.422	-.245
	70s	-.706	.302	.409	-1.625	.213
	80s	-.606	.722	1.000	-2.805	1.592
30s	20s	.599*	.102	.000	.288	.910
	40s	-.436*	.108	.001	-.765	-.107
	50s	-.628*	.127	.000	-1.015	-.241
	60s	-.234	.194	1.000	-.825	.357
	70s	-.107	.302	1.000	-1.028	.814
	80s	-.007	.722	1.000	-2.206	2.192
40s	20s	1.035*	.107	.000	.711	1.360
	30s	.436*	.108	.001	.107	.765
	50s	-.192	.131	1.000	-.590	.206
	60s	.202	.196	1.000	-.396	.800
	70s	.329	.304	1.000	-.596	1.254
	80s	.429	.723	1.000	-1.772	2.630
50s	20s	1.227*	.126	.000	.844	1.610
	30s	.628*	.127	.000	.241	1.015
	40s	.192	.131	1.000	-.206	.590
	60s	.394	.207	1.000	-.238	1.025
	70s	.521	.311	1.000	-.426	1.468
	80s	.621	.726	1.000	-1.590	2.831
60s	20s	.833*	.193	.000	.245	1.422
	30s	.234	.194	1.000	-.357	.825
	40s	-.202	.196	1.000	-.800	.396
	50s	-.394	.207	1.000	-1.025	.238
	70s	.127	.344	1.000	-.920	1.174
	80s	.227	.740	1.000	-2.028	2.482
70s	20s	.706	.302	.409	-.213	1.625
	30s	.107	.302	1.000	-.814	1.028
	40s	-.329	.304	1.000	-1.254	.596
	50s	-.521	.311	1.000	-1.468	.426
	60s	-.127	.344	1.000	-1.174	.920
	80s	.100	.776	1.000	-2.263	2.463
80s	20s	.606	.722	1.000	-1.592	2.805
	30s	.007	.722	1.000	-2.192	2.206
	40s	-.429	.723	1.000	-2.630	1.772
	50s	-.621	.726	1.000	-2.831	1.590
	60s	-.227	.740	1.000	-2.482	2.028
	70s	-.100	.776	1.000	-2.463	2.263

* The mean difference is significant at the .05 level.

Dependent Variable is CTR, yrs = years.

**Table 5:** Cross comparison of TCD among age groups of females

(I) Age (yrs)	(J) Age (yrs)	Mean Difference (I-J)	Std. Error	*p*-value	95% Confidence Interval for Difference	

					Lower Bound	Upper Bound
20s	30s	-.683*	.100	.000	-.987	-.378
	40s	-1.210*	.106	.000	-1.534	-.886
	50s	-1.470*	.112	.000	-1.810	-1.130
	60s	-1.474*	.128	.000	-1.865	-1.084
	70s	-.789*	.208	.003	-1.423	-.155
	80s	-1.481	.555	.164	-3.172	.211
30s	20s	.683*	.100	.000	.378	.987
	40s	-.527*	.115	.000	-.878	-.177
	50s	-.787*	.120	.000	-1.152	-.422
	60s	-.792*	.135	.000	-1.204	-.379
	70s	-.106	.213	1.000	-.753	.542
	80s	-.798	.557	1.000	-2.494	.899
40s	20s	1.210*	.106	.000	.886	1.534
	30s	.527*	.115	.000	.177	.878
	50s	-.260	.125	.806	-.641	.122
	60s	-.264	.140	1.000	-.692	.163
	70s	.422	.216	1.000	-.236	1.079
	80s	-.270	.558	1.000	-1.971	1.430
50s	20s	1.470*	.112	.000	1.130	1.810
	30s	.787*	.120	.000	.422	1.152
	40s	.260	.125	.806	-.122	.641
	60s	-.005	.144	1.000	-.444	.435
	70s	.681*	.218	.039	.016	1.346
	80s	-.011	.559	1.000	-1.714	1.693
60s	20s	1.474*	.128	.000	1.084	1.865
	30s	.792*	.135	.000	.379	1.204
	40s	.264	.140	1.000	-.163	.692
	50s	.005	.144	1.000	-.435	.444
	70s	.686	.227	.055	-.007	1.378
	80s	-.006	.563	1.000	-1.720	1.708
70s	20s	.789*	.208	.003	.155	1.423
	30s	.106	.213	1.000	-.542	.753
	40s	-.422	.216	1.000	-1.079	.236
	50s	-.681*	.218	.039	-1.346	-.016
	60s	-.686	.227	.055	-1.378	.007
	80s	-.692	.586	1.000	-2.477	1.093
80s	20s	1.481	.555	.164	-.211	3.172
	30s	.798	.557	1.000	-.899	2.494
	40s	.270	.558	1.000	-1.430	1.971
	50s	.011	.559	1.000	-1.693	1.714
	60s	.006	.563	1.000	-1.708	1.720
	70s	.692	.586	1.000	-1.093	2.477

* The mean difference is significant at the .05 level.

Dependent Variable is CTR, yrs = years

Table 2 and 3 show that there was no significant difference between the 80–89 year group and any other group. In males, the CTR values of the 20–29, and 30–39 year groups vary significantly (pvalue < 0.05) with the 40–49 and 50–59 year groups. In Tables 4 and 5, there was no significant difference between the 80–89 year group and any other group. In males, the frequency of significant differences among age groups decreases as the patients ages increase. Hence, the 20–29 year group differs significantly (p-value < 0.05) from the 30–39, 40–49, 50–59, 60–69 year groups, whereas the 60–69 year group only varies significantly with the 20–29 year group. The females show a similar trend as the males with a decreasing frequency of significant differences among age groups with increasing age, but as with Table 3, there are more significant differences among the female age groups than observed in the male age groups. The 20–29 year group differs significantly from the 30–39, 40–49, 50–59, 60–69 and 70–79 year groups.

## Discussion

Minor differences in transverse cardiac diameter over a short period of time should alert a physician to the possibility of worsening heart disease (3,4). We deem it important that cardiac measurements meant to detect cardiomegaly need to be very accurate. This ensures prompt referral to the cardiologist for further evaluation, often with more expensive and sophisticated tests such as echocardiography, coronary calcium scoring and computer tomographic or conventional coronary angiography. Early referral allows for early treatment, especially in resource poor communities (13). There is currently no Ghanaian study stating the existence of significant gender or age-related differences in cardiac size parameters obtained from routine, frontal chest radiographs. Ogunmodede et al in a study conducted in Nigeria recorded significant relationships between cardiac measurements and age, as differences of the measurements within ethnic groups and regions (14). This study included findings from the radiological reports of 2004 individuals, with ages ranging from 20–86 years, and reports significant gender and age-related differences in cardiac size parameters obtained from routine, frontal chest radiographs.

The mean age for the study was 39.4 ± 14.04 years. The data sets consisted of 1,053 (53.0%) males and 951 (47.0%) females.

This study demonstrates that the average CTR and TCD values were 46.6% ± 3.7 and 14.0cm ± 1.3 for males, and 47.7% ±3.7 and 12.9 ± 1.2 for females. These figures are slightly higher than those recorded by Mensah et al, and Oladipo et al whose studies involved the general populace (15). The CTR figure for males is, however, similar to 46% recorded for adult African/West Indies males by Nicole et al (16). As observed in the study, there were significant gender differences in CTR from the 30–39 years group to the 60–69 years group. However, no significant differences were noted in young adults aged 20–29 years and in the elderly above the age of 70 years. Significant gender differences were also noted in the TCD of all age groups, except the 80–89 years group (however the number of patients in that group is too small to provide a good statistical projection). This finding is supported by data recoded by Oberman et al (17). Available data considers a 1.5cm to 2cm increase in the TCD measured on consecutive posteroanterior chest radiographs taken at short intervals apart as a sign of worsening cardiac pathology, which often presents as cardiomegaly (CTR > 50%) (3,4). It has, however, been suggested that because of variations due to cardiac filling and phase of respiration, a margin of safety of 2% above the upper limit needs to be added (18). An earlier study by the authors showed that one cardiac size parameter value may not be appropriate for the whole population. They argued that due to differences in TCD and CTR averages in different age groups, different TCD increases, less than 1.5cm, were needed to record a CTR of 50%. That study showed that a minimum increase of 0.8mm for males aged 61– 80 years, and a maximum increase of 1.4cm for males aged 21–40 years resulted in a CTR of 50%. A TCD increase of 1.5cm in males aged 61–80 years would result in a CTR value of 52.2%. This would delay treatment which could have been started at a CTR of 50%, had a 0.8cm difference in TCD value been considered significant for this age group (9). This position is supported by the study by Nicole et al (16) which stated that it was unsatisfactory to have a single upper limit value for cardiothoracic ratio, such as the 50% in current use.

This study supports our earlier study (9) and that by Mihara et al (19) by demonstrating significant differences in CTR and TCD among different age groups of both sexes. We also noted no significant difference between the 80–89 year group and any other group, however once again, the number of patients in that group is too small to provide a good statistical projection. In males, the CTR values of the 20–29, and 30–39 year groups vary significantly with the 40–49 and 50–59 year groups. The elderly males, 60 years and older, however, had no significant differences with the other age groups, hence can use the normal CTR values of either young adults or middle-aged adults in the determination of cardiomegaly. In females, more significant differences were noted between the various age groups. The 20–29, and 30–39 year groups vary significantly with the 40–49, 50–59, 60–69 and 70–79 year groups. No significant differences were noted among the 40 – 49, 50 – 59, 60 – 69 and 70–79 year groups.

When the various TCDs were compared among the age groups for males and females, there was no significant difference between the 80–89 year group and any other group. In males, the frequency of significant differences among age groups decreases as the patients ages increase. Hence, the 20–29 year group differs significantly from the 30–39, 40–49, 50–59, 60–69 year groups, whereas the 60–69 year group only varies significantly with the 20–29 year group. The females show a similar trend as the males with a decreasing frequency of significant differences among age groups with increasing age, but, there are more significant differences among the female age groups than observed in the male age groups. The 20–29 year group differs significantly from the 30–39, 40–49, 50–59, 60–69 and 70–79 year groups.

The study also showed that CTR, TCD and TTD increased statistically with age of patients. It seems, as stated by other authors, recognition of the significant parametric differences could positively affect the management of cardiovascular disease (CVD), especially in resource poor countries like Ghana, where noncommunicable diseases (NCD) were estimated to account for 43% of all deaths in 2016 (20). Of the deaths due to NCD in 2016, CVD accounted for 19% of deaths making the early and accurate detection of CVD an important issue for health workers and patients (20).

In summary, the study reveals that there are significant gender and age-related differences in cardiac size parameters obtained from routine, frontal chest radiographs. These differences, if considered during patient management, may result in early and appropriate treatment of cardiac pathology in some age groups.
